# Prominent Hypercoagulability Associated With Inflammatory State Among Cancer Patients With SARS-CoV-2 Infection

**DOI:** 10.3389/fonc.2020.01345

**Published:** 2020-07-16

**Authors:** Bei Xiong, Tao Liu, Ping Luo, Yongchang Wei, Yi Zhou, Minghui Liu, Yongxi Zhang, Hanlun Wang, Xiaochun Zhang, Xinghuan Wang, Fuling Zhou

**Affiliations:** ^1^Department of Hematology, Zhongnan Hospital of Wuhan University, Wuhan, China; ^2^Department of Urology, Center for Evidence-Based and Translational Medicine, Zhongnan Hospital of Wuhan University, Wuhan, China; ^3^Department of Radiation Oncology, Zhongnan Hospital of Wuhan University, Wuhan, China; ^4^Department of Infection Disease, Zhongnan Hospital of Wuhan University, Wuhan, China; ^5^Department of Radiology, Zhongnan Hospital of Wuhan University, Wuhan, China

**Keywords:** SARS-CoV-2, COVID-19, cancer, D-dimer, hypercoagulability

## Abstract

Abnormal coagulation parameters and potential benefits of anticoagulant therapy in general population with novel coronavirus pneumonia (COVID-19) have been reported. However, limited data are available on cancer patients. Coagulation indexes and inflammation parameters in 57 cancer patients with SARS-CoV-2 infection with different severity were retrospectively analyzed. We found that D-dimer levels were increased in 33 patients (57.9%, median: 790 ng/mL). Compared with ordinary type patients, severe and critical ill patients had decreased MPV values (*P* = 0.006), prolonged PT (median: 13.3 vs. 11.5 vs. 11.4 s, *P* < 0.001), significant higher D-dimer levels (median: 2,400 vs. 940 vs. 280 ng/mL, *P* < 0.001), higher PCT levels (median: 0.17 vs. 0.055 vs. 0.045 ng/mL, *P* = 0.002), higher IL-6 (median: 20.6 vs. 2.3 vs. 3.0 pg/mL, *P* = 0.040), and decreased PaO_2_ (median: 68 vs. 84 vs. 96 mm Hg, *P* < 0.001). Importantly, three patients, one severe and two critical ill type, with increased D-dimer survived after anticoagulant therapy with continuous heparin infusion. Increased D-dimer levels positively correlated with increased PCT levels (*r* = 0.456, *P* = 0.002) and IL-6 levels (*r* = 0.501, *P* = 0.045). A negative correlation between D-dimer levels and PaO_2_ levels (*r* = −0.654, *P* = 0.021) were also existed. Cancer patients with COVID-19 showed prominent hypercoagulability associated with severe inflammation, anticoagulation therapy might be useful to improve the prognosis and should be immediately used after the onset of hypercoagulability.

## Introduction

Currently, severe acute respiratory syndrome coronavirus 2 (SARS-CoV-2) has caused pandemic outbreak all over the world. The 2019 novel coronavirus is an enveloped non-segmented positive-strand RNA virus and belongs to the genus β (1, 2). The novel coronavirus is transmitted to humans and is generally considered to cause infection through infected human respiratory droplets and contact (3–6).

The children and infants usually have mild and self-limited courses, while elderly and those with chronic diseases usually experience serious infection (4). It is reported that cancer patients harbored a higher risk of SARS-CoV-2 infection and had poorer outcomes than patients without cancer (7, 8). Researches have validated that abnormal coagulation parameters are closely associated with severity of coronavirus disease 2019 (COVID-19) (9–11), and anticoagulants can reduce mortality of COVID patients (12). However, details of coagulation state and its association with the severity of disease especially inflammation state in cancer patients with SARS-CoV-2 infection remain poorly understood. Hypercoagulability is a derangement of hemostasis characterized by increased D-dimer. Hypercoagulable disorders have a well-known widespread association with venous thromboembolic disease (13). Considering the high incidence and damage of cancer-related hypercoagulability, we investigated the clinical significance of coagulation indexes and related parameters and the role of anticoagulant therapy in cancer patients with SARS-CoV-2 infection for the first time in this study.

## Methods

### Study Design and Participants

As of March 21th, 2020, 57 cancer patients with confirmed COVID-19 were identified at Zhongnan Hospital and the Medical Treatment Alliance of Wuhan University. Meanwhile, 141 non-cancer patients with COVID-19 cases had also been identified as a control from Zhongnan Hospital of Wuhan University. All patients met the diagnostic criteria of “Diagnosis and Treatment Scheme of Novel Coronavirus–Infected Pneumonia (trial version 6)” formulated by National Health Commission of the People's Republic of China (14). According to the clinical diagnostic standard, the ordinary case had fever, respiratory symptoms, and radiographic manifestations of pneumonia; the severe case was made if patients met any of the following criteria: (1) respiratory rate ≥30 breaths/min; (2) SpO_2_ ≤ 93% while breathing room air; (3) PaO_2_/FiO_2_ ≤ 300 mmHg, or the lesion of chest CT progressing more than 50% within 24–48 h; the critically ill case was diagnosed if any of the following criteria was met: (1) respiratory failure which requiring mechanical ventilation; (2) shock; (3) combined with other organ failure, admitted to ICU. We performed a retrospective review of the medical records of these patients to collect data on medical history, comorbidities, clinical and laboratory findings, treatment, and outcomes. The durations from onset of disease to hospital admission, dyspnea, ARDS, and ICU admission were included. This case series was approved by the institutional ethics board of Zhongnan Hospital of Wuhan University (No. 2020021).

### Real-Time Reverse Transcription Polymerase Chain Reaction Assay

Throat swab samples were collected for extracting RNA from cancer patients suspected of having COVID-19. All samples were tested for SARS-CoV-2 by use of qRT-PCR with the CDC recommended Kit (BioGerm). The test results were confirmed by nested RT-PCR with designed primers. Partial S segment sequences (nt 21730-22458) were amplified with primers: 5′-CTCAGGACTTGTTCTTACCTT-3′ and 5′-CAAGTGCACAGTCTAC-AGC-3′.

### The Detection of Coagulation Indexes and Related Parameters

Here, 2 mL of blood samples were centrifuged at 3,000 r/min for 10 min and the plasma was immediately separated. Coagulation indexes, including prothrombin time (PT), activated partial thromboplastin time (APTT), plasma fibrinogen (FIB) and D-dimer levels were detected by CA-7000 automatic blood coagulation analyzer. Additionally, 1 mL of blood samples using ethylenediamine tetraacetic acid as anti-coagulant were analyzed for platelet (PLT) count, mean platelet volume (MPV) and lymphocytes by Beckman-Coulter LH750 automatic hematology analyzer. Serum C-reactive protein (CRP) levels were measured using immune transmission turbidity method by ADVIA2400 automatic chemistry analyzer. Procalcitonin (PCT) and interleukin-6 (IL-6) were detected by Mini Vidas automatic fluorescence immunoassay analyzer. The levels of arterial oxygen (PaO_2_) were also detected by automatic blood gas analyzer.

### Statistical Analysis

Continuous variables were directly expressed as a range. Categorical variables were expressed as number (%). Numerical variables are reported as mean or median [interquartile range (IQR)]. Continuous variables among different groups were compared using independent group *t*-tests when the data were normally distributed; otherwise, the Mann-Whitney *U*-test was used. Pearson correlation was used to analyze the association between coagulation indexes and related parameters. *P* < 0.05 was considered to indicate statistical significance. All statistical analyses were performed using SPSS 21.0 software (SPSS Inc).

## Results

### Clinical Characteristics

Fifty-seven cancer patients with COVID-19 were included in our study. Clinical characteristics, treatment, and outcomes of the 57 cancer patients with COVID-19 are listed in Table 1. Of the total, one patient with bladder cancer with post-chemotherapy myelosuppression and one patient with postoperative rectal cancer were presumed to have acquired infection through hospital-related transmission, while the other patients had a history of epidemiological exposure to COVID-19. 94.7% patients received empirical antibiotic therapy. Most of them received antiviral therapy (63.2%). Thirteen patients (22.8%) who required mechanical ventilation were admitted to intensive care unit (ICU), and nine patients (15.8%) received anticoagulation therapy (heparin). As of March 21th, 2020, 9 patients died, 34 were discharged to their homes with an average hospital stay of 22 days (range: 12–55 days), and the remaining were still hospitalized.

**Table 1:** Based clinical characteristics of 57 COVID-19 patients with malignancy.

**Characteristics**	**No. (%)**
**No. of patients**	57
**Age**	66 (56.5–71.5)
**Sex**	
Male	35 (61.4%)
Female	22 (38.6%)
**Comorbidity**	
Diabetes	13 (22.8%)
Hypertension	21 (36.8%)
Cardiovascular disease	11 (19.3%)
COPD	4 (7.0%)
**Symptoms and signs**	
Fever	46 (80.7%)
Cough	44 (77.2%)
Fatigue	43 (75.4%)
Shortness of breath	28 (49.1%)
Headache	8 (14.0%)
Nausea and vomiting	5 (8.8%)
**Bilateral pneumonia**	54 (94.7%)
**Major complications**	
Respiratory Distress Syndrome	19 (33.3%)
Arrhythmia	8 (14.0%)
Septic shock	5 (8.7%)
Heart failure	13 (22.8%)
**Severity of illness**	
General	19 (33.3%)
Severe	19 (33.3%)
Critical ill	19 (33.3%)
**Treatment**	
Mechanical ventilation	13 (22.8%)
Antiviral therapy	36 (63.2%)
Antibiotic therapy	54 (94.7%)
Use of corticosteroid	24 (42.1%)
Intravenous immunoglobin	21 (36.8%)
Anti-coagulation therapy	9 (15.8%)
**Clinical outcome**	
Hospitalization	14 (24.6%)
Discharged	34 (59.6%)
Death	9 (15.8%)

*COPD, Chronic obstructive pulmonary disease*.

### Assessment of Coagulation Indexes

Table 2 presented the coagulation index and other parameters in cancer patients. 57.9% of these patients had an increased level of D-dimer (>500 ng/mL). All nine non-survivors showed increased D-dimer. Twenty-one patients (36.84%) had prolonged PT despite median levels for these patients being in the normal range (median: 11.6 s, IQR: 11.1–13.2 s). In addition, normal FIB (median: 332 mg/dL, IQR: 291-407 mg/dL). and APTT (median: 28.3 s, IQR: 25.4–32.4 s) were observed. None of the patients presented with disseminated intravascular coagulation according to the Consensus of Chinese Experts on the Diagnosis of Disseminated Intravascular Coagulation (version 2017) (15). To have a better understanding of the coagulation profile, we compared the coagulation index between COVID-19 patients with cancer and without cancer. Compared with non-cancer patients, cancer patients with COVID-19 presented relatively higher MPV (median 10.2 vs. 9.1 fL, *P* < 0.001), decreased APTT (median 28.3 vs. 30.0 s, *P* = 0.047), decreased FIB (median 332 vs. 427.5 mg/dL, *P* < 0.001), and significantly increased D-dimer levels (median 790 vs. 262 ng/mL, *P* < 0.001) (Table S1).

**Table 2:** Coagulation index and related parameter among different severity COVID-19 patients with malignancy.

**Item**	**Total (*N* = 57)**	**Ordinary type (*N* = 19)**	**Severe type (*N* = 19)**	**Critical ill type (*N* = 19)**	***P*-value**
PLT, × 10^9^/L (NR: 125–350)	200 (100–264.5)	235 (165–266)	188 (128–234)	142.5 (61.3–293.5)	0.212
Increased No. (%)	4/56^†^ (7.1%)	1/18^†^ (5.6%)	0	3 (15.8%)	
Decreased No. (%)	14/56^†^ (25%)	3/18^†^ (16.7%)	4 (21.1%)	7 (36.8%)	
MPV, fl (NR: 6–12)	10.2 (9.7–11.3)	10.1 (9.5–10.4)	10.9 (10.3–11.9)	9.7 (8.6–11.5)	0.006
Increased No. (%)	3 (5.3%)	0	3 (15.8%)	0	
Decreased No. (%)	0	0	0	0	
PT, S (NR: 9.4–12.5)	11.6 (11.1–13.2)	11.4 (10.8–11.8)	11.5 (10.9–11.9)	13.3 (12.3–15.2)	<0.001
Increased No. (%)	21 (36.84%)	4 (21.05%)	4 (21.05%)	13 (76.47%)	
Decreased No. (%)	0	0	0	0	
APTT, S (NR: 25.1–36.5)	28.3 (25.4–32.4)	28.3 (24.8–31.1)	27.8 (25.2–33.4)	29.4 (26.0–32.2)	0.873
Increased No. (%)	4 (7.0%)	1 (5.2%)	1 (5.2%)	2 (10.5%)	
Decreased No. (%)	12 (21.1%)	6 (31.6%)	4 (21.1%)	2 (10.5%)	
FIB, mg/dL (NR: 238–498)	332 (291–407)	365 (295–419)	318 (290–388)	352 (271–430)	0.664
Increased No. (%)	6 (10.5%)	1 (5.2%)	2 (10.5%)	3 (15.8%)	
Decreased No. (%)	7 (12.3%)	1 (5.2%)	2 (10.5%)	4 (21.1%)	
D-dimer, ng/mL (NR: 0–500)	790 (330–1,890)	280 (184.5–385)	940 (570–1,890)	2,400 (1,006–12,722)	<0.001
Increased No. (%)	33 (57.9%)	1 (5.2%)	15 (78.9%)	17 (89.5%)	
CRP, mg/L (NR: 0–10)	37.6 (9.5–103.4)	12.7 (0.85–25.4)	49.4 (25.8–59.8)	101.7 (10.8–172)	0.140
Increased No. (%)	44 (77.2%)	13 (68.4%)	15 (78.9%)	16 (84.2%)	
PCT, ng/ml (NR: < 0.05)	0.06 (0.03–0.17)	0.045 (0.03–0.06)	0.055 (0.03–0.125)	0.17 (0.09–0.429)	0.002
Increased No. (%)	24 (42.1%)	5 (26.3%)	7 (36.8%)	12 (63.2%)	
IL-6, pg/ml (NR: 0–7)	3.5 (1.5–10.3)	3.0 (1.6–3.9)	2.3 (1.5–10.3)	20.6 (3.6–67.5)	0.040
Increased No. (%)	37 (64.9%)	7 (36.8%)	16 (84.2%)	14 (73.7%)	
PaO_2_, mm Hg (NR: 83–108)	89 (74–95)	96 (95–98)	84 (79–90)	68 (62–72)	<0.001
Decreased No. (%)	24 (42.1%)	0 (%)	5 (26.3%)	19 (100%)	

*NR, normal range; IQR, interquartile range; PLT, platelet count; MPV, mean platelet volume; PT, prothrombin time; APTT, activated partial thromboplastin time; FIB, fibrinogen; CRP, C-reactive protein; PCT, procalcitonin; IL-6, interleukin-6. Data are presented as Median (IQR)*.

† *One patient with bladder cancer at the myelosuppression stage was excluded*.

### Evaluation of Related Parameters

To have a better understanding of the coagulation function in cancer patients with COVID-19, we evaluated some other related parameters. One bladder cancer case was excluded from the statistical analysis for PLT counts owing to its low PLT count caused by post-chemotherapy myelosuppression. Fourteen of the remaining 56 patients (25%) had low PLT counts, and the median PLT count in the 56 patients was 200 × 10^9^/L (IQR: 100–264.5 × 10^9^/L). Three patients (5.3%) presented with increased MPV values. 44 (77.2%) patients showed increased CRP (median: 37.6 mg/L, IQR: 9.5–103.4 mg/L). PCT levels were elevated in 24 (42.1%) patients (median: 0.06 ng/mL, IQR: 0.03–0.17 ng/mL), IL-6 levels were increased in 37 (64.9%) patients (median: 3.5 pg/mL, IQR: 1.5–10.3 pg/mL), and PaO_2_ were decreased in 24 (42.1%) patients (median: 89 mm Hg, IQR: 74–95 mm Hg) (Table 2).

### Comparison Among Groups

In order to further evaluate coagulation indexes after initial coronavirus infection, we divided patients into three groups according to severity of illness for COVID-19 patients with cancer. Compared with ordinary type patients, severe and critical ill patients had decreased MPV values (*P* = 0.006), prolonged PT (median: 13.3 vs. 11.5 vs. 11.4 s, *P* < 0.001), significant higher D-dimer levels (median: 2,400 vs. 940 vs. 280 ng/mL, *P* < 0.001), higher PCT levels (median: 0.17 vs. 0.055 vs. 0.045 ng/mL, *P* = 0.002), higher IL-6 (median: 20.6 vs. 2.3 vs. 3.0 pg/mL, *P* = 0.040), and decreased PaO_2_ (median: 68 vs. 84 vs. 96 mm Hg, *P* < 0.001) (Table 2).

### Correlation Analysis

To find the possible reason of the hypercoagulability in cancer patients with COVID-19, we analyzed the association of coagulation indexes and platelet parameters with the levels of CRP, PCT, IL-6, lymphocytes and PaO_2_ (Table 3). The results revealed a positive correlation between D-dimer levels and PCT levels (*r* = 0.456, *P* = 0.002) and IL-6 levels (*r* = 0.501, *P* = 0.045). A positive correlation between PCT levels and PT were also found (*r* = 0.401, *P* = 0.008). A negative correlation between lymphocytes and PT levels (*r* = −0.522, *P* < 0.001) as well as D-dimer (*r* = −0.354, *P* = 0.009) were observed. D-dimer levels were found to be negative correlated with PaO_2_ levels (*r* = −0.654, *P* = 0.021). We also found a positive correlation between PLT counts and lymphocytes (*r* = 0.371, *P* = 0.005).

**Table 3:** Correlation analysis of coagulation indexes and related parameters in 57 COVID-19 patients with malignancy.

**Index**	**Correlation coefficient**	**CRP, mg/L**	**PCT, ng/ml**	**IL-6, pg/ml**	**Lymphocytes, × 10^**9**^/L**	**PaO_**2**_, mm Hg**
PT, S	*r*	0.293	0.401	0.229	−0.522	−0.401
	*P*	0.310	0.008	0.270	<0.001	0.038
APTT, S	*r*	0.450	0.001	0.321	−0.255	−0.175
	*P*	0.106	0.994	0.118	0.058	0.408
FIB, mg/dL	*r*	0.087	0.110	0.195	−0.158	0.209
	*P*	0.767	0.485	0.351	0.245	0.338
D–dimer, ng/mL	*r*	0.070	0.456	0.501	−0.354	−0.654
	*P*	0.820	0.002	0.045	0.009	0.021
PLT, × 10^9^/L	*r*	0.031	−0.241	−0.246	0.371	0.057
	*P*	0.919	0.120	0.236	0.005	0.709
MPV, fL	*r*	0.331	−0.257	−0.127	−0.075	−0.135
	*P*	0.294	0.131	0.555	0.629	0.503

*CRP, C-reactive protein; PCT, procalcitonin; IL-6, interleukin-6; PT, prothrombin time; APTT, activated partial thromboplastin time; FIB, fibrinogen; PLT, platelet count; MPV, mean platelet volume*.

### Anticoagulation Therapy

Three patients, one severe and two critical ill type, with increased D-dimer who accepted anticoagulant therapy with low molecular weight heparin (LMWH) survived after starting. The cancer status of all these three patients are in indolent disease states.

**Figure 1:**
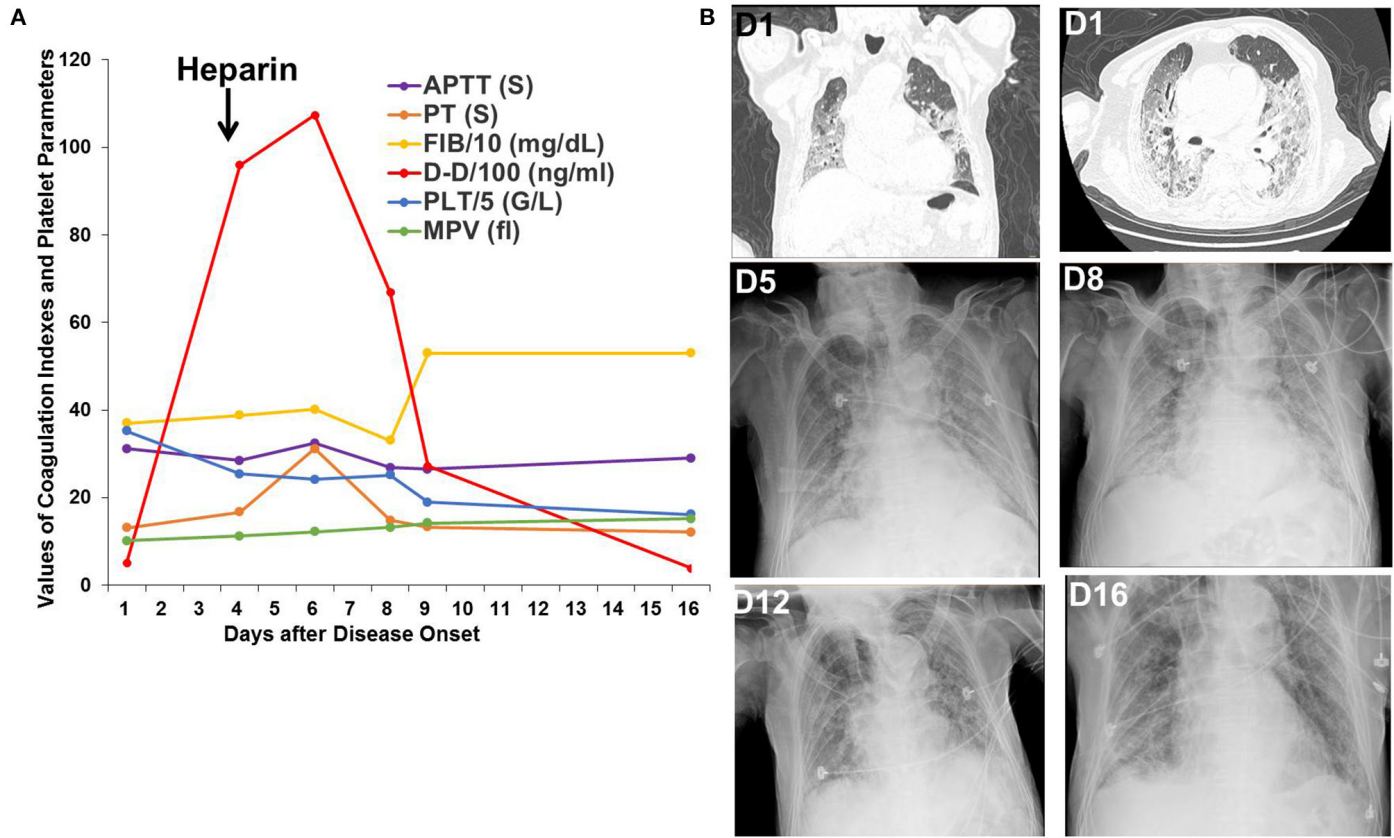
Changes in coagulation indexes, platelet parameters, chest CT, and X-ray images in Patient 1. **(A)** Changes in coagulation indexes and platelet parameters showed a decrease in D-dimer levels after heparin treatment. **(B)** Chest CT and X-ray images showed improvement in the multiple, bilateral, ground-glass opacities.

Patient 1 was an 87-year-old patient with gastric cancer who received surgery in 2009 and did not receive chemotherapy or radiotherapy for gastric cancer. He was admitted to the hospital after having fever for 10 days and chest pain, shortness of breath for 5 days. His SpO_2_ was only 70%, and his chest CT showed multiple, bilateral, ground-glass opacities on admission. His D-dimer level was 515 ng/mL on the first day and increased to 9,596 ng/mL on the fourth day. Heparin was administrated beginning on the fourth day after disease onset. After non-invasive ventilation, glucocorticoids, heparin, antibiotic treatment, and antiviral therapy in the ICU, his SpO_2_ remained above 95%, and his pulmonary lesions were gradually absorbed. His D-dimer level decreased to 386 ng/mL on the sixteenth day (Figure 1).

**Figure 2:**
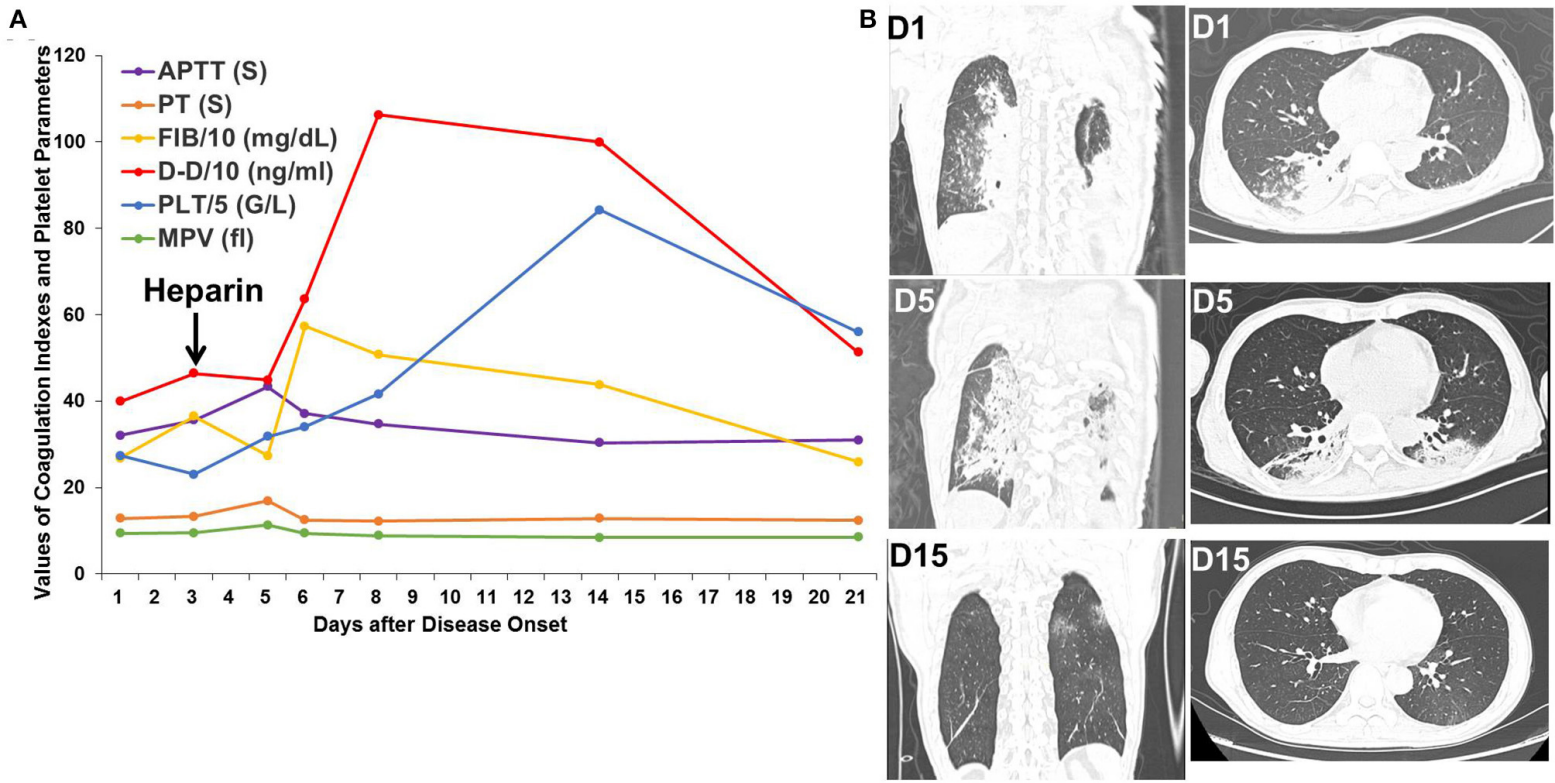
Changes in coagulation indexes, platelet parameters, and chest CT scans in Patient 2. **(A)** Changes in coagulation indexes and platelet parameters showed an initial increase followed by a decrease in D-dimer levels after heparin treatment. **(B)** Chest CT scans showed improvement in the bilateral, ground-glass opacities.

Patient 2 was a 54-year-old patient with rectal cancer who received surgery for rectal cancer on Jan 16, 2020. An intermittent, postoperative fever lead to a chest CT scan, which showed bilateral, ground-glass opacities. SpO_2_ remained at 95–99% throughout the clinical course. D-dimer increased from 399 ng/mL on the first day to 464 ng/mL on the third day and peaked at 1,062 ng/mL on the eighth day. Heparin was administered from the third day after disease onset. After administration of heparin, antibiotic treatment, and supportive care, significant absorption of the pulmonary bilateral, ground-glass opacities was observed, as was decreased D-dimer to 513 ng/mL on the twenty-first day (Figure 2).

**Figure 3:**
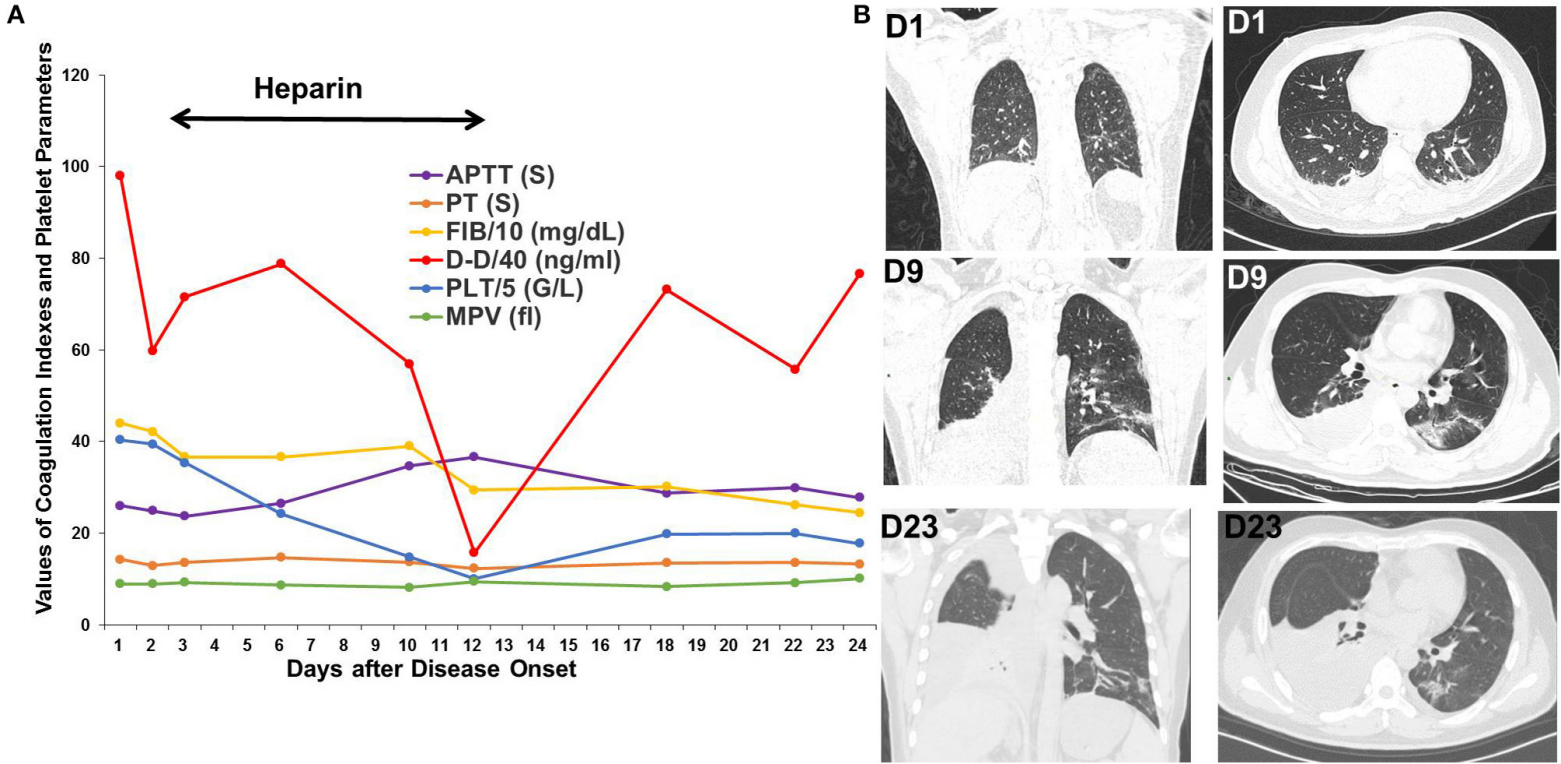
Changes in coagulation indexes, platelet parameters and chest CT scans in Patient 3. **(A)** Changes in coagulation indexes and platelet parameters showed a decrease in D-dimer levels after using heparin but a rebound after its withdrawal. **(B)** CT scans showed significant improvement in the bilateral, ground-glass opacities but persistent hydrothorax on the right.

Patient 3 was a 37-year-old patient with hepatic cancer who received surgery 1 month prior to being admitted for fever, nausea, and vomiting for 2 days. The D-dimer level was 3,919 ng/mL upon admission. Chest CT scan showed several bilateral slivers on the lower lobes; a small amount of hydrothorax evolving into bilateral, ground-glass opacities; and increased hydrothorax on the right. Heparin was administered from the third day after disease onset and withdrawed due to decreased platelet count to 50 G/L on the twelfth day. After oxygen therapy, glucocorticoids, heparin, antibiotic treatment, and antiviral therapy in the ICU, the bilateral, ground-glass opacities improved, but the hydrothorax persisted on the right side. D-dimer decreased to 630 ng/mL on the twelfth day but rebounded to a high level after withdrawal of heparin (Figure 3).

## Discussion

The SARS-CoV-2 outbreak began in December 2019 in Wuhan, Hubei province, and rapidly spread to many provinces in China as well as to other countries. COVID-19 is a highly infectious disease with a long incubation period, and it has a variety of clinical manifestations, which has a significant impact on public health and life. Severe cases of COVID-19 and mortality are significantly higher among the elderly than among other age groups (9, 16, 17). Because of impaired immunity, influence of radiotherapy or chemotherapy, elder age and comorbidities, severe and critical ill pneumonia are more frequently seen in cancer patients than in general population with COVID (7, 8). The mortality of cancer patients with COVID (15.8%) in this study was much higher than the general population with COVID (4.3–15%) (9, 16, 17), but lower than the mortality reported in COVID-19 patients with thoracic malignancies (18). The discrepancy of mortality in cancer patients between our and Leora's study may be explained by the geographical locations. Most patients included in Leora's study were from European (Italy, France, and Spain). All of these regions were particularly hard hit. Here, we evaluated coagulation functions and related parameters and the potential effect of anticoagulation therapy in cancer patients with SARS-CoV-2 infection for the first time and aimed to improve the prognosis of patients with cancer.

Our results showed that cancer patients with COVID-19 had prominent increased D-dimer levels. More importantly, increased D-dimer levels were closely related to the severity and mortality of the disease. This finding is in accordance with the results in general populations with COVID (9, 10, 19). However, cancer patients not only showed a much higher rate of increased D-dimer but also significantly elevated D-dimer levels compared with non-cancer patients. Therefore, the level of D-dimer tends to be more important in reflecting the prognosis of COVID-19 in cancer patients.

In our study, we found that 21 patients had mildly prolonged PT. APTT and FIB didn't change much. These changes of fibrinogen, PT and APTT in cancer patients are similar with most reports in general population with COVID-19 (9, 10, 16, 17). Although decreased PLT counts were observed in 25% of cancer patients, the extent of PLT decrease is not pronounced. Also, there had been DIC appearing in none of cancer patients. This is different from what Tang's findings that the existence of DIC was common in the deaths of general populations with COVID-19 (11). Therefore, in our studies, cancer patients exhibited a prominent hypercoagulability characterized by increased D-dimer, but did not show the consumption coagulopathy as characterized by significant reduced platelet counts, low fibrinogen and prolonged PT/APTT.

The exact reasons for the observed hypercoagulability in cancer patients with COVID-19 are unknown. Hypercoagulability could be a common complication in cancer. Increased procoagulants such as tissue factor, elevated platelet activity, damaged endothelium and blood flow abnormalities may all contribute to hypercoagulability in cancer patients (20). Cancer patients with poor performance status or receiving several medications such as hormonal therapy or chemotherapy also contributed to risk of hypercoagulability (21). These aspects of cancer-related hypercoagulability have been known for a long time. Since emerging data increasingly suggest that severe COVID-19 reflects dysregulated inflammation and it has been repeatedly shown that inflammation could produce a hypercoagulable state (12), here we investigated whether the hypercoagulability in cancer patients with COVID-19 were associated with inflammation state. We found that cancer patients with severe and critical ill type showed higher PCT and IL-6 levels than ordinary type, suggesting that overproduction of early response proinflammatory cytokines leading to poor prognosis. More importantly, our results showed that D-dimer levels positively correlated with increased PCT and IL-6 levels, adding evidence that severe inflammation associated with hypercoagulability in cancer patients with COVID-19. Cytokines and inflammatory mediators due to inflammatory reaction to SARS-Cov-2 caused damage in endothelial cells of pulmonary blood vessels and peripheral blood vessels. The inflammatory reaction also leads to the activation of the coagulation and fibrinolysis systems (22, 23).

This hypercoagulability could contribute to the development of life-threatening pulmonary embolism. It has been reported recently that a certain number of patients with COVID-19 showed clinical evidences of pulmonary embolism (24). Autopsies performed on critical patient with COVID-19 showed occlusion and microthrombosis formation in pulmonary small vessels (25). Pulmonary embolism diagnoses are also supported by the fact that up to 1/3 of COVID-19 patients had deep vein thromboses of the lower limbs (26). At the early time of COVID-19 outbreak, the administration of anticoagulant therapy has not been widely used for fear of potential bleeding risk in severe patients and previous negative trials of endogenous anticoagulants in sepsis, but now more evidences have supported the use of prophylactic dose LMWH in hospitalized patients with COVID-19 to prevent venous thromboembolism (12, 27). Although the diagnosis of pulmonary embolism in our studies could not be confirmed by CT angiography or autopsy, massive D-dimer elevations and the association between increased D-dimer levels and decreased PaO_2_ in these patients highly indicated the existence of pulmonary embolism. Three patients in our study who received LMWH showed significantly reductions in raised D-dimer concentrations as well as improvement of pulmonary lesions. LMWH may not only quench the hypercoagulability state and possibly venous thromboembolism, but may also improve the severe inflammation state through its anti-inflammatory effect in these patients.

Our studies have several limitations. First, this is a retrospective study and we couldn't choose the potential candidate to be administrated with LMWH. Second, most of the baseline data of coagulation parameters in these cancer patients before having SARS-Cov-2 infection were not available to have a better idea of the coagulation changes in cancer patients with COVID-19 because of limited available data. Third, there were no direct evidence of pulmonary embolism or deep vein thromboses in these patients to help fully understand the correlations of D-dimer, hypercoagulability, thromboembolism, and inflammation in cancer patients with COVID-19. Fourth, the number of patients enrolled in our study is limited. Nevertheless, our results indicated that prominent hypercoagulability are associated with severe inflammation in cancer patients and anticoagulant therapy in cancer patients with COVID-19 are effective. Coagulation indexes should be closely monitored during treatment. Large-scale studies are needed to confirm our findings and further investigations are still essential to identify proper patients to benefit from heparin treat as well as the time point to intervene.

## Data Availability Statement

All datasets presented in this study are included in the article/Supplementary Material.

## Ethics Statement

The studies involving human participants were reviewed and approved by the Institutional Ethics Board of Zhongnan Hospital of Wuhan University (No. 2020021). Written informed consent for participation was not required for this study in accordance with the national legislation and the institutional requirements.

## Author Contributions

PL, BX, TL, and FZ conceived and designed the study, take responsibility for the integrity of the data, and the accuracy of the data analysis. YW, YZho, ML, YZha, HW, and XZ collected, analyzed, and interpreted the data. BX and PL performed the statistical analysis and wrote the manuscript. FZ and XW revised the manuscript. All authors reviewed and approved the final version.

## Conflict of Interest

The authors declare that the research was conducted in the absence of any commercial or financial relationships that could be construed as a potential conflict of interest.
