# Supplementary material for: Prominent Hypercoagulability Associated With Inflammatory State Among Cancer Patients With SARS-CoV-2 Infection
Source: Front Oncol. 2020 Jul 16;10:1345. doi: 10.3389/fonc.2020.01345 (PMC7378817; doi:10.3389/fonc.2020.01345)
Supplement: Supplementary file 1 [file Table_1.docx]

**Table S1** **Comparison of Coagulation Index between COVID-19 Patients with Malignancy and without Malignancy**

| **Item** | **Group A (n=57)**  Median (IQR) | **Group B (n=141)**  Median (IQR) | ***P* value^a^** |
| --- | --- | --- | --- |
| PLT, ×10^9^/L | 200 (119.75-264.5) | 181 (139.5-227.5) | 0.703 |
| Increased No. (%) | 14/56† (25.0%) | 3 (2.1%) |  |
| Decreased No. (%) | 4/56† (7.1%) | 25 (17.7%) |  |
| MPV, fL | 10.2 (9.65-11.3) | 9.10 (8.15-10.20) | ＜0.001 |
| Increased No. (%) | 3 (5.3%) | 2 (1.4%) |  |
| Decreased No. (%) | 0 | 0 |  |
| PT, S | 11.6 (11.1-13.2) | 12.7 (11.4-13.5) | 0.079 |
| Increased No. (%) | 21 (36.8%) | 77 (54.6%) |  |
| Decreased No. (%) | 0 | 2 (1.4%) |  |
| APTT, S | 28.3 (25.4-32.4) | 30.0 (27.8-32.4) | 0.047 |
| Increased No. (%) | 4 (7.0%) | 2 (1.4%) |  |
| Decreased No. (%) | 12 (21.1%) | 11 (7.8%) |  |
| FIB, mg/dL | 332 (291-407) | 427.5 (383.8-492) | ＜0.001 |
| Increased No. (%) | 6 (10.5%) | 33 (23.4%) |  |
| Decreased No. (%) | 7 (12.3%) | 2 (1.4%) |  |
| D-dimer, ng/mL | 790 (330-1890) | 262(146-589) | ＜0.001 |
| Increased No. (%) | 33 (57.9%) | 39 (27.7%) |  |

Abbreviations: IQR, interquartile range; PLT, platelet count; MPV, mean platelet volume; PT, prothrombin time; APTT, activated partial thromboplastin time; FIB, fibrinogen.

^†^One patient with bladder cancer at the myelosuppression stage was excluded.

Group A: COVID-19 patients with malignancy, Group B: COVID-19 patients without malignancy
